# *Danshen* Polysaccharides Alleviate Aflatoxin B1-Induced Liver Damage and Immune Disorders by Inhibiting the ROS-Mediated Mitochondrial Apoptosis Pathway

**DOI:** 10.3390/antiox14080991

**Published:** 2025-08-13

**Authors:** Lu Zhang, Yongzhan Bao, Xincheng Gong, Shuang Ma, Xiao Wang, Wanyu Shi

**Affiliations:** 1 College of Traditional Chinese Veterinary Medicine, Hebei Agricultural University, No. 2596 Lekai South Street, Baoding 071000, China; zhanglu1323@126.com (L.Z.); baoyongzhan2006@126.com (Y.B.); gongxin6328@sina.com (X.G.); 2 School of Life Science and Food Engineering, Hebei University of Engineering, Handan 056000, China; mashuang@hebeu.edu.cn; 3 Hebei Key Laboratory of Traditional Chinese Veterinary Medicine, Baoding 071001, China

**Keywords:** aflatoxin B1, *Danshen* polysaccharide, liver damage, immune dysfunction

## Abstract

*Danshen* polysaccharide (DSPS) is the main natural compound extracted from the traditional Chinese herb *Danshen*. Although DSPS is well-known for its antioxidant and anti-inflammatory properties, its impact on aflatoxin B1 (AFB1)-induced damage has not been explored. This study aims to investigate the potential protective mechanisms of DSPS against AFB1-induced liver damage and immune disorders. The experiment lasted a total of three weeks, during which 120 rabbits were randomly assigned to six groups (*n* = 20). AFB1 and DSPS were incorporated into the diets of each group. We found that DSPS significantly inhibited AFB1-induced hepatocyte edema, inflammatory cell infiltration, and increased serum aspartate aminotransferase (AST)/ alanine aminotransferase (ALT) levels (*p* < 0.05). DSPS alleviated oxidative damage by downregulating CYP1A1/A2 mRNA, enhancing liver total antioxidant capacity (T-AOC), superoxide dismutase (SOD), and glutathione (GSH) levels, and reducing the production of reactive oxygen species (ROS) and malondialdehyde (MDA) (*p* < 0.05). DSPS inhibits the expression of cytochrome c (cyt.c), caspase 9, and caspase 3, significantly reducing the apoptosis rate of hepatocytes (*p* < 0.05). Additionally, DSPS elevates the levels of immunoglobulins (IgA, IgG, IgM) and interferon-gamma (IFN-γ), while decreasing the concentration of IL-4 (*p* < 0.05). This study demonstrates that DSPS can alleviate AFB1-induced damage, with the underlying mechanisms likely related to enhanced antioxidant capacity, inhibition of oxidative stress, and intrinsic apoptotic pathways, as well as improved immune responses.

## 1. Introduction

In humid and hot regions, the contamination of aflatoxin B1 (AFB1), a highly toxic mycotoxin produced by Aspergillus flavus, is particularly prominent. Grains such as corn and rice are highly susceptible to AFB1 contamination when exposed to environments with a water activity of 0.91–0.99 aw and temperatures ranging from 25 to 37 °C [1]. Once AFB1 enters the body through the food chain, its unique bisfuran ring structure demonstrates significant toxicological differences during first-pass metabolism in the liver [2]. The CYP450 enzymes in the liver catalyze the epoxidation of AFB1, resulting in the formation of the highly reactive AFB1-8,9-epoxide (AFBO). AFBO can covalently bind to DNA and proteins, leading to DNA damage in hepatocytes [3]. Furthermore, AFB1 exposure can induce excessive production of reactive oxygen species (ROS), disrupting the intracellular redox balance of glutathione (GSH) and oxidized glutathione (GSSG). This disruption leads to mitochondrial membrane potential depolarization, which subsequently activates the caspase cascade and ultimately induces apoptosis in hepatocytes [4]. Research has confirmed that AFB1 can significantly increase the proportion of apoptotic cells in rabbit liver tissue, reaching up to 5.7 times the normal levels [5]. The immunotoxicity of AFB1 exhibits a bidirectional characteristic that is dependent on dose, exposure duration, and species [6]. As a crucial organ of the innate immune system, the liver harbors a dense population of immune cells [7]. However, exposure to AFB1 can impair the immune surveillance function of the liver, particularly by disrupting the activity of Kupffer cells and natural killer (NK) cells [8]. In vitro, in vivo, and ex vivo experiments have confirmed that AFB1 can enhance pyroptosis of hepatocytes and activate Kupffer cells by promoting the dephosphorylation of cyclooxygenase-2, thereby exacerbating liver damage [9]. Exposure to AFB1 can lead to a significant increase in the number of immune cells, such as monocytes, macrophages, lymphocytes, and dendritic cells [10]. Additionally, AFB1 can suppress the synthesis of immunoglobulins, hinder the proliferation of T and B lymphocytes, and enhance the susceptibility of the body to pathogens [11].

*Danshen* is a widely utilized medicinal plant, particularly for treating cardiovascular diseases and diabetes [12]. This plant possesses antioxidant and immunomodulatory properties and is often taken as a health supplement [13,14]. *Danshen* polysaccharide (DSPS), the main active component extracted from *Danshen*, shares similar pharmacological effects [15,16]. Research indicates that DSPS significantly reduces LPS-induced immune-mediated liver damage in mice by lowering liver and immune organ indices and by inhibiting aspartate aminotransferase (AST), alanine aminotransferase (ALT), and NO levels [17]. DSPS improves the functionality of antioxidant enzymes, reduces lipid peroxidation products, and inhibits ferroptosis [16]. Furthermore, DSPS can enhance macrophage respiratory burst and promote lymphocyte proliferation, highlighting its significant immunomodulatory potential [13,18].

## 2. Materials and Methods

### 2.1. Animals and Experimental Design

One hundred and twenty New Zealand meat rabbits (0.69 ± 0.04 kg; 35 days old) were purchased from a commercial breeding farm in Baoding, China. Following a 5-day acclimation period, the rabbits were allocated at random into six groups: the control group (GA), the model group (GB), the low-dose DSPS group (GC), the medium-dose DSPS group (GD), the high-dose DSPS group (GE), and the DSPS group (GF). Each rabbit was housed individually in wire rabbit cages (60 × 55 × 45 cm—w × d × h), where they were provided ad libitum access to water and feed. The feed composition is consistent with previous studies [5]. The dietary interventions for each group are outlined in Table 1. The experimental phase lasted 3 weeks. The composition and nutritional levels of the experimental diet are shown in Table S1.

### 2.2. Biochemical Indicators

Serum was separated according to standard protocols, and a 10% liver homogenate was prepared [19,20]. Commercial assay kits (Nanjing Jiancheng Research Institute, Nanjing, China) were used to measure the levels of AST and ALT in the serum, as well as total antioxidant capacity (T-AOC), superoxide dismutase (SOD), malondialdehyde (MDA), and GSH in the liver. A fully automated hematology analyzer (BC-5000vet, Mindray International Medical Company, Shenzhen, China) was employed to determine the counts of white blood cells (WBC), lymphocytes (Lymph), granulocytes (MON), and neutrophil granulocytes (Gran) in the blood samples from each group of rabbits.

### 2.3. ELISA

The levels of serum immunoglobulin (IgA, IgG, IgM) and cytokines (IL-2, IL-4, IL-6, IFN-γ) were measured using their respective ELISA kits (Enzyme-linked Biology Co., Ltd, Shanghai, China).

### 2.4. The Fluorescence Probe Method (DCFH-DA)

The liver samples were sliced into 5 μm frozen sections. The sections were incubated separately with ROS and DAPI (Beyotime Biotechnology Co., Ltd, Shanghai, China) staining solutions in the dark. The sections were then washed with PBS and mounted with an anti-fluorescence quencher. DAPI was used to label the nuclei, rendering them blue, while ROS dye was employed to indicate the levels of ROS, appearing green [5]. Fluorescence microscopy images were analyzed with ImageJ software (version 1.41o, Bethesda, MD, USA).

### 2.5. Histopathological Analysis and TUNEL Reaction

For histological analysis, liver samples were fixed in 4% paraformaldehyde and sliced into 5 µm thick sections by a microtome. Samples were subsequently immersed in hematoxylin for 3 min, followed by thorough rinsing with double-distilled water and then with tap water. They were then stained with eosin for 15 s and rinsed again. After removing excess liquid by air drying, an appropriate amount of neutral resin was added, and the slides were sealed and allowed to dry for storage.

Paraffin sections underwent a rigorous deparaffinization protocol, followed by treatment with proteinase K working solution for 20 min to expose the DNA break ends. Residual proteinase and fixative in the sections were removed using PBS wash buffer. The Tunel reaction mixture was incubated with the tissue at 37 °C for 1 h, allowing the TdT enzyme to catalyze the incorporation of labeled dUTP at the DNA strand breaks. Subsequently, the tissue was then exposed to DAPI staining solution for 10 min [3].

### 2.6. QPCR and Western Blotting

Total RNA was isolated from frozen liver tissue. The spectrophotometer (ND-1000, NanoDrop Technologies, Wilmington, DE, USA) was used to evaluate the purity of RNA at an optical ratio of OD260/OD280 in the range of 1.9 and 2.1. The extracted RNA was then reverse-transcribed into cDNA. The resulting cDNA was diluted to the optimal concentration and amplified by real-time quantitative PCR [21,22]. The primer sequences for PCR are shown in Table S2.

Liver tissue was lysed in radio-immuno-precipitation assay buffer, separated using SDS-PAGE, and transferred to a membrane. After blocking in 5% low-fat milk solution, the membranes were then incubated with primary antibodies overnight at 4 °C. Membranes were incubated at room temperature with a horseradish peroxidase-linked secondary antibody for 2 h. Chemiluminescence was used to visualize immune complexes [23]. Protein bands were quantified using ImageJ software. The specific antibody information for Western blotting is shown in Table S3.

### 2.7. Statistical Analyses

All statistical analyses were performed using SPSS 19.0 software, with one-way ANOVA and Tukey’s multiple comparison tests. The results of the statistics are presented in terms of the mean ± standard deviation (SD).

## 3. Results

### 3.1. The Effects of DSPS on Growth Performance

The mortality rate in the model group of meat rabbits was three times higher than that in the GA group, while the mortality rate in the GE group was comparable to that in the GA group. Compared to the GA group, the model group exhibited a 45.49% reduction in ADG and a 30.26% decrease in ADFI, while the FCR increased by 28.22% (*p* < 0.05). In contrast, the GE group showed a 36.84% improvement in ADG, a 22.94% increase in ADFI, and a 10.03% decrease in FCR relative to the model group (*p* < 0.05). Additionally, the ADFI in the GF group was 9.69% higher than that of the control group (*p* < 0.05) (Table 2).

### 3.2. The Effects of DSPS on Liver Function

**Figure 1:**
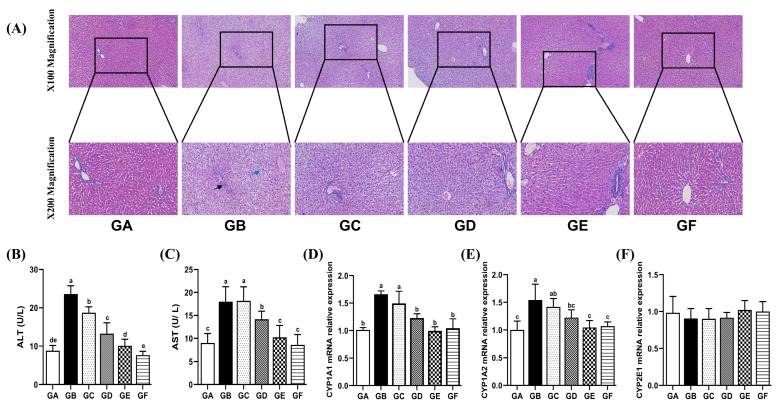
Effects of AFB1 and DSPS administration on liver function. (**A**) Representative images of H&E-stained liver sections. Blue arrows indicate hepatocyte edema; black arrows denote inflammatory cell infiltration, scale bar: 100 or 50 μm. (**B**,**C**) The serum of ALT and AST levels. (**D**–**F**) Relative mRNA levels of CYP1A1, CYP1A2, and CYP2E1 in the liver. Note: *p* < 0.05 was considered indicative of statistical significance. Significant differences exist between different letter labels, and no significant differences exist between the same letter labels, as shown below.

Hepatocytes in the model group exhibited edema, distorted or absent nuclear structures, and a marked infiltration of inflammatory cells within the liver parenchyma, in contrast to those in the control group. However, a notable improvement in this condition was observed with increasing doses of DSPS (Figure 1A).

In the model group, serum levels of AST and ALT were significantly elevated compared to those in the GA group (*p* < 0.05) (Figure 1B,C). However, both medium and high doses of DSPS significantly reduced AST and ALT levels compared to the model group (*p* < 0.05). Furthermore, the AST and ALT levels of the GE group were similar to those of the GA group, with no notable variations between them.

### 3.3. The Effects of DSPS on Drug Metabolizing Enzymes

AFB1 resulted in a notable elevation in the relative expression levels of CYP1A1 and CYP1A2 mRNA (*p* < 0.05). In the GE group, there were no significant differences in the transcription levels of CYP1A1 and CYP1A2 compared to the control group. Furthermore, the expression of CYP2E1 mRNA did not show significant differences among the groups (Figure 1D–F).

### 3.4. The Effects of DSPS on Antioxidant Capacity

**Figure 2:**
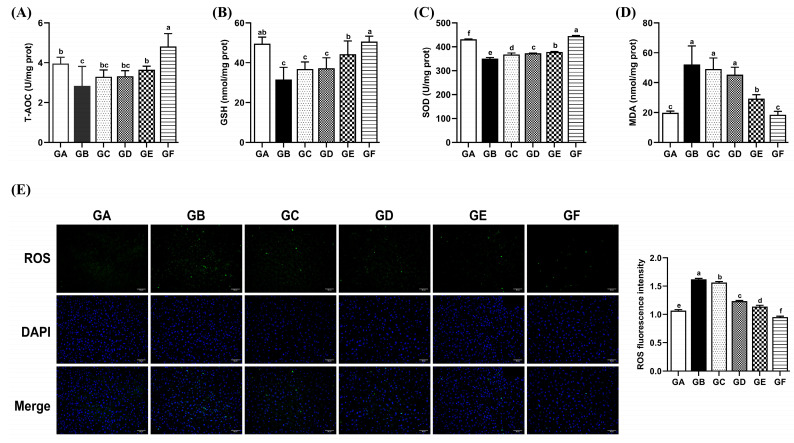
Effects of AFB1 and DSPS administration on liver antioxidant capacity. (**A**–**D**) The activities of T-AOC, GSH, SOD, and MDA in the liver. (**E**) Fluorescence images of livers stained with ROS (magnification × 200; scale bar 50 μm).

Compared to the control group, the model group exhibited significantly reduced levels of T-AOC, SOD, and GSH in the liver (*p* < 0.05), accompanied by significantly increased levels of MDA and ROS (*p* < 0.05). The GE group showed significant increases in T-AOC, SOD, and GSH levels (*p* < 0.05), along with significant decreases in MDA and ROS levels compared to the model group (*p* < 0.05). Moreover, the GF group demonstrated significantly higher levels of T-AOC and SOD than the control group (*p* < 0.05), while MDA and ROS levels were significantly lower than those in the control group (*p* < 0.05) (Figure 2A–E).

### 3.5. The Effects of DSPS on Hepatocyte Apoptosis

**Figure 3:**
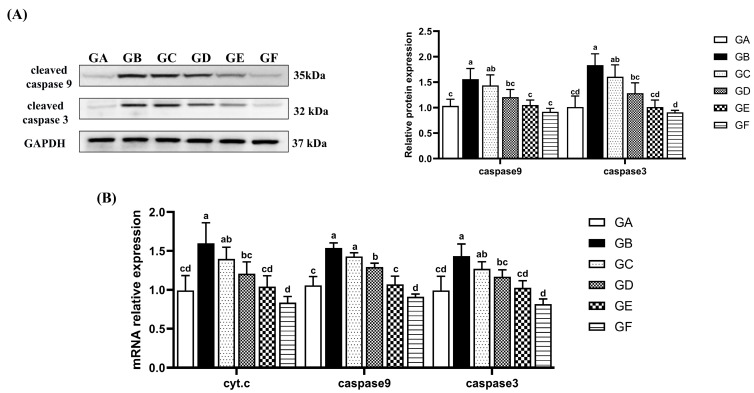
Effects of AFB1 and DSPS administration on mitochondrial apoptosis pathway markers. (**A**) The protein abundance of cleaved caspase 9 and cleaved caspase 3 in liver. (**B**) The mRNA levels of cyt.c, caspase 9, and caspase 3 in liver.

In the model group, the relative expression levels of cyt.c, caspase 9, and caspase 3 mRNA in the liver were significantly higher than those in the control group (*p* < 0.05). In the GE group, the transcription levels of cyt.c, caspase 9, and caspase 3 were not only significantly lower than those in the model group (*p* < 0.05), but did not differ significantly from the control group. Additionally, the protein abundance of caspase 9 and caspase 3 showed a trend consistent with their transcription levels (Figure 3A,B).

**Figure 4:**
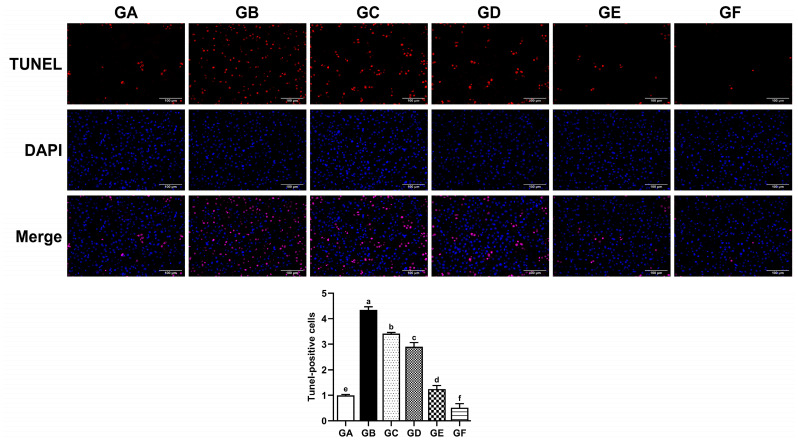
Effects of AFB1 and DSPS administration on the apoptosis rate of liver cells (magnification × 200; scale bar 100 μm).

Compared to the control group, the liver apoptosis rate in the model group was significantly increased (*p* < 0.05), while the apoptosis rate in the GF group was significantly decreased (*p* < 0.05). The apoptosis rates in the DSPS treatment groups were significantly lower than those in the model group (*p* < 0.05) (Figure 4).

### 3.6. The Effects of DSPS on the Immune Organ Index

**Figure 5:**
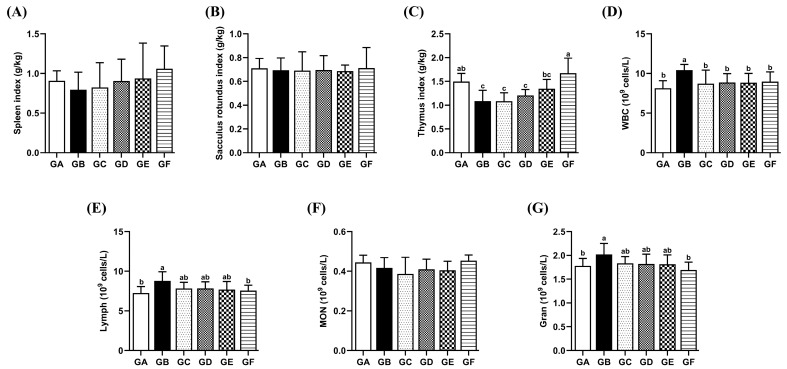
Effects of AFB1 and DSPS administration on immune organ index, white blood cell, and its subpopulation numbers. (**A**–**C**) Spleen, sacculus rotundus, and thymic indices. (**D**–**G**) The number of WBC, Lymph, MON, and Gran in the blood.

The thymus index in the model group rabbits was markedly reduced compared to the control group (*p* < 0.05). The thymus index in the GE group did not show a significant difference compared to the control group. There were no significant differences in spleen index and round lymphatic follicle index among the treatment groups (Figure 5A–C).

### 3.7. The Effects of DSPS on the Quantity of Leukocytes and Their Subpopulations

The levels of WBC, Lymph, and Gran in the blood of the model group were significantly higher than those in the control group (*p* < 0.05). The WBC count in all three DSPS treatment groups exhibited a statistically significant decrease compared to that of the model group (*p* < 0.05). No statistically significant variation was observed in the cell counts when comparing the DSPS-treated groups with the control group. Additionally, the monocyte count in each group showed no significant difference (Figure 5D–G).

### 3.8. The Effects of DSPS on Immunoglobulins

**Figure 6:**
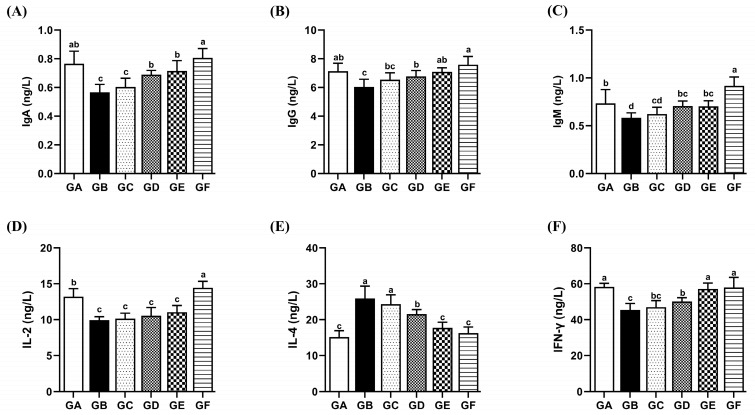
Effects of AFB1 and DSPS administration on immunoglobulin and immune cytokines. (**A**–**C**) The levels of IgA, IgG, and IgM in serum. (**D**–**F**) The levels of IL-2, IL-4, and IFN-γ in serum.

In the model group, there was a notable reduction in the serum concentrations of IgA, IgG, and IgM (*p* < 0.05). Following intervention with medium and high doses of the drug, the levels of IgA, IgG, and IgM significantly increased (*p* < 0.05). Furthermore, the immunoglobulin levels in the GE group did not differ significantly from those in the control group (Figure 6A–C).

### 3.9. The Effects of DSPS on Immune Cell Cytokines

In the model group, the levels of IL-2 and IFN-γ were significantly decreased (*p* < 0.05), while IL-4 levels were significantly increased compared to the control group (*p* < 0.05). In contrast, the GE group showed a significant increase in IFN-γ levels and a significant decrease in IL-4 levels compared to the model group (*p* < 0.05) (Figure 6D–F).

## 4. Discussion

AFB1 is the most toxic among various aflatoxins, inducing liver damage and disrupting the host immune response [20,24]. Previous studies have demonstrated that dietary AFB1 supplementation leads to hepatocyte swelling, vacuolization, and portal fibrosis in piglets [25]. Additionally, AFB1 exposure causes ballooning degeneration, steatosis, and nuclear condensation in mouse hepatocytes [26]. In meat rabbits exposed to AFB1, we observed hepatocellular edema, nuclear dissolution, and infiltration of inflammatory cells. Furthermore, AFB1 exposure significantly increased serum AST and ALT activities, while DSPS treatment markedly alleviated these pathological changes in the liver. These findings suggest that DSPS effectively mitigates AFB1-induced liver damage in meat rabbits.

The CYP 450 family serves as a pivotal hub for the biotransformation/bioactivation of AFB1 in the liver [27]. It catalyzes the oxidative reactions of AFB1, generating active intermediate metabolites with increased toxicity and carcinogenicity, thereby playing a crucial role in its carcinogenesis process [27]. In vivo and in vitro experiments have confirmed that AFB1 can significantly increase the transcriptional levels of CYP1A1, CYP1A2, and CYP3A6 mRNAs in rat liver tissues and human cells [28]. Consistent with previous studies [29,30], this study showed that AFB1 could induce an increase in the expression of CYP1A1 and CYP1A2 mRNAs. However, dietary supplementation with 900 μg/kg DSPS could inhibit their expression. Different CYP enzymes may exhibit differences in their binding capacity and metabolic efficiency toward AFB1 [31]. AFB1 exhibited significant negative binding energy with CYP1A1 and CYP1A2, indicating strong and stable binding capacity [31]. Although CYP2E1 is involved in the metabolism of xenobiotics and procarcinogens, it is not the major cytochrome enzyme for AFB1 metabolism [32]. Thus, AFB1 showed no significant effect on CYP2E1 in this study.

AFB1-induced ROS accumulation disrupts the intracellular redox balance, leading to an increase in products such as lipid peroxidation [3,33]. Additionally, GSH, an important detoxifying enzyme for AFB1, binds to AFBO to form AFB1-GSH conjugates, neutralizing AFB1 toxicity [34,35]. However, excessive consumption of GSH further impairs the body’s antioxidant capacity. The results of this study showed that after AFB1 treatment, the levels of hepatic antioxidant enzymes (GSH, SOD) and T-AOC were significantly decreased, while the contents of lipid peroxidation products (MDA) and ROS were significantly increased. Dietary DSPS effectively alleviated AFB1-induced oxidative stress. AFB1 exposure increased the sensitivity of hepatocytes to oxidative stress, leading to cell death [36]. Under continuous stimulation by ROS, mitochondria release cytochrome c, which binds to apoptotic protease activating factor-1 and pro-caspase-9 to form the apoptosome complex, thereby promoting the activation of downstream effector caspase 3 [3,37]. The supplementation of AFB1 in broiler diets was found to induce excessive production of ROS and MDA in the liver, accompanied by decreased activities of GSH, CAT, and SOD, as well as downregulated mRNA expression of Bax and caspase 3 [38]. Wang et al. confirmed that AFB1 inhibits SOD and GST, induces hepatic oxidative stress, thereby upregulating the expression of p53, Bax, cyt.c, caspase-9, and caspase-3 while downregulating Bcl-2 expression, ultimately triggering the intrinsic apoptotic pathway [39]. In this study, dietary supplementation with DSPS inhibited AFB1-induced expression of cyt.c, caspase 9, and caspase 3 and reduced the hepatic cell apoptosis rate. These findings indicate that the reduction in hepatocyte apoptosis by DSPS may be associated with the inhibition of intrinsic apoptotic pathway activation.

The survival and function of immune cells are regulated by redox homeostasis, with their activity dependent on the dynamic balance between intracellular/extracellular ROS and reactive nitrogen species [40]. AFB1 can inhibit T-cell activation, resulting in insufficient secretion of IL-2 and IFN-γ, which, in turn, hinders the differentiation of B cells into plasma cells, leading to impaired synthesis of antibodies such as IgG and IgM [41]. Meanwhile, oxidatively modified TCR receptors impair the ability of T cells to recognize antigens [42]. Consistent with our results, continuous supplementation of AFB1 in broiler diets for 21 days significantly increased the levels of WBC, Mon, and Gran in serum, while significantly decreasing the percentages of T-cell subsets and the contents of IL-2 and IFN-γ in serum [41]. T cells are the target cells of DSPS [43]. DSPS can promote the proliferation of T lymphocytes in cancer patients by regulating the expression of cytokines (such as IL-2, IL-4, IL-6, IFN-γ) [44]. This study found that dietary supplementation with 900 μg/kg DSPS increased the levels of IgA, IgG, and IgM, which enhances humoral immune response capacity. Meanwhile, DSPS can also inhibit the secretion of Th1-type cytokine (IFN-γ) and upregulate the expression of Th2-type cytokine (IL-4). These changes are related to the improvement in immune parameters.

This study has certain limitations. Firstly, the observed association between DSPS and the reduction in AFB1-induced damage does not establish a direct causal relationship, as causal experiments were not conducted. Secondly, this research was carried out in a single animal model (New Zealand rabbit); thus, the efficacy in other species, including humans, remains unclear. Lastly, while our study did not reveal any adverse effects from DSPS supplementation alone, it is crucial to evaluate the long-term safety and dose-dependent effects of DSPS, as these factors are essential for its potential practical applications.

## 5. Conclusions

In summary, AFB1 triggers oxidative stress through cytochrome metabolism, accelerating hepatocyte apoptosis and impairing immune function. In contrast, dietary supplementation with DSPS exhibits potential protective properties, which are associated with alleviated oxidative stress, reduced hepatocyte apoptosis, and enhanced immune function.

## Figures and Tables

**Table 1:** The content of mycotoxins and drugs in rabbit diet.

Items	GA	GB	GC	GD	GE	GF
AFB1 (μg/kg)	ND	25	25	25	25	ND
DON/ZEA	ND	ND	ND	ND	ND	ND
ZEA	ND	ND	ND	ND	ND	ND
DSPS (mg/kg)	ND	ND	300	600	900	900

**Table 2:** Effects of DSPS on the growth performance and apparent digestibility of rabbits.

Items	GA	GB	GC	GD	GE	GF
Mortality (%)	5	15	15	10	5	0
ADG (g/d)	47.46 ± 2.03 ^b^	25.87 ± 1.40 ^e^	29.37 ± 2.46 ^e^	33.33 ± 0.85 ^d^	35.40 ± 1.40 ^c^	52.06 ± 1.87 ^a^
ADFI (g/d)	114.60 ± 6.72 ^a^	79.92 ± 9.09 ^c^	81.27 ± 4.84 ^c^	92.86 ± 6.75 ^b^	98.25 ± 3.38 ^b^	115.24 ± 5.04 ^a^
FCR	2.41 ± 0.06 ^c^	3.09 ± 0.28 ^a^	2.78 ± 0.33 ^a^	2.78 ± 0.14 ^b^	2.78 ± 0.10 ^b^	2.21 ± 0.06 ^c^

Note: Significant differences exist between different letter labels, and no significant differences exist between the same letter labels, as shown below.

## Data Availability

Data will be made available upon request.

## Supplementary Materials

The following supporting information can be downloaded at https://www.mdpi.com/article/10.3390/antiox14080991/s1, Table S1: Composition and nutrition level of experimental diet; Table S2. Primer sequences used for Real-time quantitative PCR; Table S3. Antibody used for Western blotting.
