# Supplementary material for: Danshen Polysaccharides Alleviate Aflatoxin B1-Induced Liver Damage and Immune Disorders by Inhibiting the ROS-Mediated Mitochondrial Apoptosis Pathway
Source: Antioxidants (Basel). 2025 Aug 13;14(8):991. doi: 10.3390/antiox14080991 (PMC12383227; doi:10.3390/antiox14080991)
Supplement: Supplementary file 1 [file antioxidants-14-00991-s001.zip › Supplementary information.pdf]

Supplementary information:

Preparation of experimental diet:

The toxigenic fungal strain used in this study was *Aspergillus flavus* strain AS3.3950, which was cultured in broth (LB) media (10 g tryptone, 5 g yeast extract, 10 g NaCl, in 1 L of distilled water) at 28 ˚C overnight. And it was titered up to a concentration of approximately 1×10^6 spores/mL with sterile water to produce a spore suspension that was used for all experiments. Spore suspension was inoculated into corn and incubated under ideal conditions (30 °C, 85% temperature) [3]. The fresh corn in the diet was then entirely substituted with corn contaminated with AFB1. DSPS (purity ≥ 95%) is incorporated into the feed formulation and processed to produce experimental rabbit feed. The molar ratios of the constituent sugars in this DSPS are 2.26:2.31:1.26:1.05:1.14 for mannose, xylose, arabinose, glucose, and galactose, respectively [5,19]. The ELISA kits were used to measure the concentrations of major mycotoxins in the feed samples from each group. The analysis revealed that the AFB1 concentration in the diet was 25 μg/kg.

Supplementary Table S1. Composition and nutrition level of experimental diet %

Ingredients	Content	Nutrient levels[2)]	
Corn	15.00	Digestive energy (MJ/kg)	10.46
Barley	12.00	Crude protein	17.00
Wheat bran	21.40	Coarse fiber	14.00
Soybean meal	8.00	Crude fat	3.00
Peanut meal	7.00	Calcium	0.85
Peanut hull	8.00	phosphorus	0.50
Chry powder	11.00	Lysine	0.80
Barley hull	15.00	Methionine + cysteine	0.60
Limestone	1.20		
Premix[1)]	0.50		

NaCl	0.50	
Met	0.20	
Lys	0.20	
Total	100.00	

[1)] Premix provided the following per kilogram of diet: Fe 70 mg, Cu 20 mg, Zn 70 mg, Mn 10 mg, Se 0.25 mg, Co 0.15 mg, I 0.2 mg, VA 10000 IU, VD 900 IU, VE 50 mg, VK 2 mg, Thiamine 2 mg, Riboflavin 6 mg, Pantothenic acid 50 mg, Pyridoxine 2 mg, VB_{12} 0.02 mg, Niacin 50 mg, Choline 1000 mg, Biotin 0.2 mg.

[2)] The nutrient level is the measured value.

Supplementary Table S2. Primer sequences used for Real-time quantitative PCR

Target gene	Primer sequence (5′–3′)	Accession number
GAPDH	F: CCACTTTGTGAAGCTCATTTCCT<br>R: TCTCGTCCTCCTCTGGTGCT	NM_001082253
CYP1A1	F: GGCCACATCCGGGACATCAC<br>R: ACACCTGGACGTTGGCATTCTC	NW_026259191.1
CYP1A2	F: GCACAGCGAAAAGAACTCCAAGG<br>R: CCAAAGATGTCGTTGACGAGGTTG	NM_001171121.1
CYP2E1	F: CATCCTCCTGTTCATATCCGTCTGG<br>R: ATGATGGGCAGTGGGAAAGGTC	XM_002718772.4
cyt-c	F: TGTTCAGAAATGTGCCCAGTG<br>R: CTTGTTGGCGTCTGTGTAGGAG	XM_002713723.4
caspase3	F: GCTGAAAGAGTACGCTCACAAACT<br>R: AAGTGGCATCAAGGGAATAGGA	NM_001082117.1
caspase9	F: TCAGGATGGTGTGAGAGGAAAC<br>R: AAGTTCACGTTGTTGATGATGAGG	XM_008249762.2

F: Forward, R: Reversed. All primers were designed and synthesized by sangon Biotechnology Co., Ltd (Shanghai, China). The gene expression was quantified using the $2^{-\Delta\Delta Ct}$ method, $\Delta\Delta Ct$ = (Ct(target, test) -Ct (reference, test)) - (Ct (target, calibrator)- Ct (reference, calibrator)).

Supplementary Table S3. Antibody used for Western blotting

Target protein	Dilution	Source	Product number
GAPDH	1:1000	Bioson Biotechnology Co., Ltd (Beijing, China)	bs-41373R
caspase-9	1:2000	Bison Biotechnology Co., Ltd (Beijing, China)	bs-20773R
caspase-3	1:2000	Abcam Trading Co., Ltd (Shanghai, China)	ab208161
HRP Goat Anti-Rabbit IgG	1:2000	Bioson Biotechnology Co., Ltd (Beijing, China)	bs-0295 G
